# Evaluating the Effect of a Novel Molluscicide in the Endemic Schistosomiasis Japonica Area of China

**DOI:** 10.3390/ijerph111010406

**Published:** 2014-10-10

**Authors:** Jing Xia, Yi Yuan, Xingjian Xu, Fenghua Wei, Guiling Li, Min Liu, Jianqiang Li, Rujuan Chen, Zhengping Zhou, Shaofa Nie

**Affiliations:** 1 Department of Epidemiology and Health Statistics, School of Public Health, Tongji Medical College, Huazhong University of Science and Technology, Wuhan 430030, China; E-Mail: xiaj0608@163.com; 2 Institute of Schistosomiasis Control, Hubei Provincial Center for Disease Control and Prevention, Wuhan 430079, China; E-Mails: yuanyi5555@foxmail.com (Y.Y.); xuxj8412@foxmail.com (X.X.); weifh9@163.com (F.W.); 3 Department of Chemistry and Chemical Industry, Huazhong University of Science and Technology, Wuhan 430030, China; E-Mails: guilingli1949@gmail.com (G.L.); Liumin@hust.edu.cn (M.L.); 4 Sichuan Chemical Industry Research and Design Institute, Chengdu 610041, China; E-Mails: hy-ljq-2008@163.com (J.L.); chenrj0808@163.com (R.C.); CYRZZP@163.com (Z.Z.)

**Keywords:** evaluation, novel molluscicide, application, schistosomiasis japonica, China

## Abstract

*Oncomelania hupensis* is the sole intermediate host snail of *Schistosoma japonicum* in China. Snail control by molluscicide remains one of the most effective measures of schistosomiasis japonica control. A 50% wettable powder of niclosamide ethanolamine salt (WPN) is widely used for snail control in China. However, WPN is costly and toxic to fish. A novel molluscicide named LDS, the salt of quinoid-2′, 5-dichloro-4′-nitrosalicylanilide from niclosamide, has been developed. To evaluate the effects of large-scale field application of LDS on field snail control, tests were conducted in 15 counties of Hubei Province, China. Active adult snails, were immersed in 0.2, 0.4, and 0.6 g/m^3^ of 10% LDS, 1.0 g/m^3^ of 50% WPN was used as the molluscicide control, and then the mortality rates of snails were investigated after 1, 2, and 3 days. In addition, four active concentrations of 10% LDS (0.4, 0.6, 0.8 and 1.0 g/m^2^) were applied by spraying and powdering in the field. 1.0 g/m^2^ of 50% WPN was used as the molluscicide control, and then the mortality rates of snails were observed after 1, 3, and 7 days. The results indicated that 0.4 g/m^3^ LDS applied by the immersion or 0.6 g/m^2^ LDS applied by spraying and powdering achieved the same molluscicidal effect as that of WPN, regardless of exposure time. By using different methods, the snail mortality rates in the molluscicide groups were related to exposure time and concentration, respectively. LDS costs less than WPN; thus, LDS is suitable and applicable for use as a molluscicide in schistosomiasis japonica epidemic areas.

## 1. Introduction

Schistosomiasis remains a major public health problem in many parts of the developing world [1]. Worldwide, almost 800 million individuals are at risk of schistosomiasis. About 200 million people are infected, over half of whom have some degree of morbidity [2]. This leads to a disease burden that might be as high as 4.5 million disability-adjusted life-years [3]. Schistosomiasis japonica is mainly prevalent in the People’s Republic of China (P.R. China), the Philippines, and small pockets of Indonesia, with P.R. China as the most heavily endemic of these three countries [4,5]. In China, schistosomiasis japonica persists in the five provinces situated around the Yangtze River Basin and its lakes, as well as in two mountainous provinces. In 2011, there were an estimated total of 286,836 cases of *S. japonicum* infection, about 372,664.10 ha of areas infested with *Oncomelania Hupensis* were found, and 1163.87 ha newly detected *O. hupensis* areas were reported [6]. The snail is widely distributed in a complex environment, resulting in challenges for *S. japonicum* control in China [6,7].

*O. hupensis* is the sole intermediate host snail of *S. japonicum* in China, extirpation of the snail makes it possible to halt the transmission dynamics of *S. japonicum* [8]. Application of chemical molluscicides is one of the most effective measures for snail control [9]. Niclosamide has been recommended by the WHO for use as a molluscicide since the 1960s and is still the molluscicide of choice [10,11]. A 50% wettable powder of niclosamide ethanolamine salt (WPN) has been widely used as a molluscicide in China [9,12,13]. Currently, 50% WPN at a dose of 1.0 mg/L is recommend by the Ministry of Health, P.R. China for snail control in the field [7]. However, WPN is costly and highly toxic to fish and other aquatic animals [14,15,16,17,18]; it therefore cannot be used in some economically poor areas and fish or crab breeding areas in China. *O. hupensis* escape from these areas after the application of WPN, thereby reducing the molluscicidal effect of the chemical [19,20,21,22].

To overcome these problems, a novel molluscicide was recently developed by derivation from niclosamide. LDS is the salt of quinoid-2′, 5-dichloro-4′-nitrosalicylanilide (10% total LDS content), which is the active ingredient of LDS and the molecular formula is a C_13_H_7_Cl_2_N_2_O_4_^-^ M^+^. The filler is Na_2_SO_4_ (89% total LDS content) and the surfactant (1% total LDS content).The fusion point of LDS is more than 300 °C, which dissolved to water and ethanol, and the solubility of LDS in the water is a 1.4 g/L. LDS is formulated as a wettable powder [23,24,25,26].

LDS has been evaluated in the laboratory and the field [23,24,25,26]. To assess the effects of large-scale field application of LDS we conducted field tests in 15 counties in schistosomiasis japonica endemic areas of Hubei Province, China. The molluscicidal effects of 10% LDS and 50% WPN were compared.

## 2. Methods

### 2.1. Study Site

**Figure 1:**
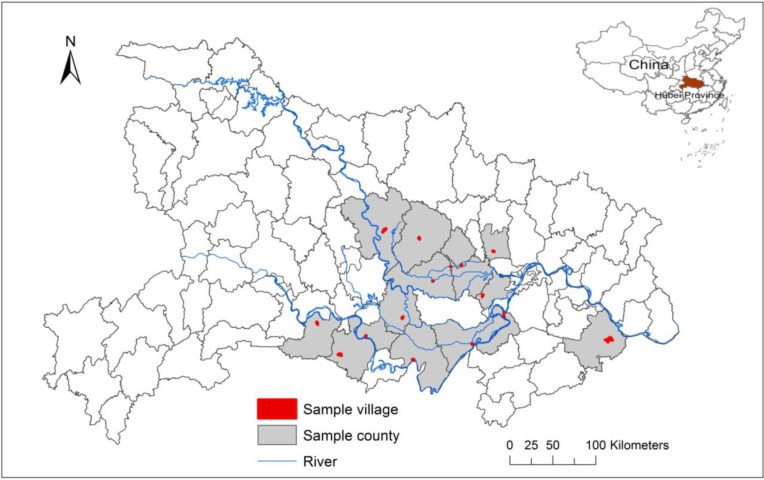
Location of the study counties, Hubei Province, China.

Hubei Province is located in the middle and lower reaches of the Yangtze River, which is an area highly endemic area for schistosomiasis in China [6,27,28]. The marshlands along the Yangtze River are dry in winter and flooded in summer, and the water levels of the marshlands are difficult to control. Most marshlands are easily flooded during the flood season, creating ideal breeding sites for *O. hupensis* [29]. In 2011, about 76,733.10 ha were infested with *O. hupensis*, and 133 ha of newly detected *O. hupensis* areas were reported in Hubei Province [6]. In the spring of 2011, the effects of LDS were tested in 15 counties in Hubei Province (Figure 1). Snail habitats in small irrigation and drainage ditches were selected for application of LDS using an immersion method and floodplains were selected to test the effects of LDS application by spraying and powdering. All testing sites in 15 counties were in identical ecological environments.

### 2.2. Molluscicide

A 10% LDS (series number 20100247), and 50% WPN (series number 2010-0-84) were obtained from Sichuan Academy of Chemical Industry and Design, Chengdu, China.

### 2.3. Molluscicidal Activity

The snails used in immersion tests were collected from the rural marshlands of 15 counties. The snails were washed with water and placed in a container with dechlorinated water. After 24 h, adult snails that climbed out of the water and displayed strong activity within four hours were selected for testing. The test was conducted in the field in the spring in 15 counties, Hubei Province. During field testing, the air temperature was between15 °C and 34 °C, the median air temperature was 21 °C to 28 °C. Small irrigation and drainage ditches near a river were selected as an immersion field, with an average snail density of 11~18/0.11 m^2^. The irrigation and drainage ditch, 200 m in length, 0.3~0.5 m in width and 0.35~0.45 m in depth, with stable water level, comprised the testing site which was divided into five equal segments separated by a 3-meter buffer. The volume of water in each segment was calculated, three active concentrations of the LDS were tested: 0.2, 0.4, and 0.6 g/m^3^ (mg/L). 1.0 g/m^3^ (mg/L) active concentration of WPN was applied as a molluscicide control. One segment was a blank control. Nine nylon bags each containing 30 snails were immersed in each segment. Three bags were removed from each section on days 1, 2, and 3. Snails collected were washed three times with dechlorinated water, than placed on a plate containing dechlorinated water and maintained for 48 h. The snails were hammered to identify whether the snails died and the numbers of snail that had died were counted [22].

The floodplain snail habitats were selected as spraying field in 15 counties, with an average snail density of 11~18/0.11 m^2^.The test sites were divided into six 100 m^2^ sections separated by a 3-meter buffer to prevent snail migration between sections. All vegetation was cut down from each section prior to the start of the experiment. The weather conditions were described above and the soil moisture content was 20%~30% in test field. Four active concentrations of LDS were tested: 0.4, 0.6, 0.8, 1.0 g/m^2^. 1.0 g/m^2^ active concentration of WPN was used as the molluscicide control. After 1, 3, and 7 days, snails were collected by a checkerboard sampling method [22]. On each sampling day, all snails within 10 sampling frames were collected from each section. The snails were tested for viability as already described above.

In the powdering test, the floodplain snail habitats were selected as testing sites, with an average snail density of 11~17/0.11 m^2^. Test sites for powdering application were prepared in the same manner as those used for tests of sprayed molluscicide. Four active concentrations of the LDS were tested: 0.4, 0.6, 0.8, 1.0 g/m^2^. 1.0 g/m^2^ active concentration of WPN was used as molluscicide control. Molluscicide for each group was mixed with sand (1:4) and the mixture was scattered over each section. After 1, 3, and 7 days, snail were collected using a checkerboard sampling method [22]. The snails were collected and tested for viability in the same way as spraying.

### 2.4. Cost Comparison

Chinese Yuan is the currency of P. R. China. The labor cost is same with when using either of the two molluscicides. The cost of WPN and LDS with the recommended concentrations was compared for each application method. We compared the cost of two molluscicides on the same cubic meter and square meter of area. The WPN cost is 38,000 CNY/ton; LDS is 12,000 CNY/ton:

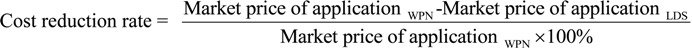


### 2.5. Statistical Analysis

Statistical analyses were performed using the Statistics Analysis System, SPSS. Comparisons between the snail mortality rates with LDS and WPN were performed using the Chi-square test. Binary logistic regression was used to analyze the effect of concentration of molluscicide and exposure time on snail mortality. *P*-values of less than 0.05 were considered to be statistically significant.

## 3. Results

### 3.1. Comparison between the Snail Mortality Rate of LDS and WPN

There was no significant difference in snail mortality in the 0.2 g/m^3^ LDS groups and WPN groups after 2, or 3 days immersion(*P* = 0.387, *P* = 0.490, Table 1). The results indicate no significant differences between the snail mortality rates at 0.4 g/m^3^ LDS and WPN after 1, or 3 days immersion (*P* = 0.442, *P* = 0.087, Table 1). There was a significant difference in snail mortality between the 0.6 g/m^3^ LDS groups and the WPN group after 1, 2, and 3 days (*P* < 0.001, *P* < 0.001, *P* = 0.005, Table 1), the snail mortality rate after exposure to 0.6 g/m^3^ LDS for 1, 2, and 3 days were higher than that of WPN (Table 1).

**Table 1:** Comparison of the adjusted mortality of snails exposed for 24–72 h to different concentrations of LDS and WPN applied by immersion in 15 counties of Hubei Province.

Molluscicide	Dose (g/m^3^)	1 day			2 days			3 days		
		Mortality Rate (%)	χ^2^	*P*-value *	Mortality Rate (%)	χ^2^	*P*-value *	Mortality Rate (%)	χ^2^	*P*-value *
LDS	0.2	83.92 (1284/1530)	6.611	0.010	94.05 (1439/1530)	0.749	0.387	98.76 (1511/1530)	0.476	0.490
	0.4	88.10 (1348/1530)	0.592	0.442	96.60 (1478/1530)	6.207	0.013	99.54 (1523/1530)	2.930	0.087
	0.6	92.22 (1411/1530)	20.982	0.000	98.50 (1507/1530)	32.642	0.000	99.80 (1527/1530)	8.047	0.005
WPN	1.0	87.19 (1334/1530)			94.77 (1450/1530)			99.02 (1515/1530)		
control	0.0	3.40 (42/1530)			4.12 (63/1530)			4.38 (67/1530)		

Note:* The mortality of snails exposed to WPN compared with that of snails exposed to three active concentrations of LDS.

There was a significant difference in snail mortality between the 0.4 g/m^2^ LDS and the WPN spraying groups after 1, 3, and 7 days (*P* = 0.002, *P* < 0.001, *P* = 0.001, Table 2).

**Table 2:** Comparison of the mortality of snails exposed for 1, 3, or 7 days to different concentrations of LDS and WPN applied by spraying and powdering in 15 counties of Hubei Province.

Molluscicide Method	Molluscicide	Dose (g/m^2^)	1 Day				3 Days				7 Days		
			Mortality Rate (%)	χ^2^	*P*-value *		Mortality Rate (%)	χ^2^	*P*-value *		Mortality Rate (%)	χ^2^	*P*-value *
spraying	LDS	0.4	73.09 (1388/1899)	9.284	0.002		82.25 (1520/1848)	15.340	0.000		88.57 (1635/1846)	12.035	0.001
		0.6	76.62 (1494/1950)	0.311	0.577		86.26 (1595/1849)	0.332	0.565		93.42 (1703/1823)	2.819	0.093
		0.8	81.41 (1546/1899)	9.399	0.002		88.13 (1589/1803)	1.244	0.265		94.15 (1707/1813)	6.705	0.010
		1.0	83.98 (1599/1904)	26.470	0.000		89.08 (1680/1886)	4.149	0.042		92.32 (1732/1876)	0.161	0.688
	WPN	1.0	77.37 (1450/1874)				86.91 (1600/1841)				91.97 (1672/1818)		
	control	0.0	4.07 (75/1843)				4.45 (85/1910)				5.64 (108/1915)		
powdering	LDS	0.4	69.41 (1305/1880)	0.012	0.912		79.18 (1453/1835)	5.304	0.021		84.30 (1664/1974)	35.590	0.000
		0.6	71.75 (1392/1940)	2.121	0.145		82.28 (1500/1823)	0.004	0.952		89.58 (1625/1814)	1.133	0.287
		0.8	77.08 (1544/2003)	27.380	0.000		84.12 (1573/1870)	2.390	0.122		92.17 (1847/2004)	3.003	0.083
		1.0	79.96 (1584/1981)	54.126	0.000		86.61 (1663/1920)	13.734	0.000		92.94 (1842/1982)	6.989	0.008
	WPN	1.0	69.58 (1249/1795)				82.20 (1469/1787)				90.62 (1758/1940)		
	control	0.0	3.79 (71/1873)				4.66 (86/1846)				5.11 (103/2016)		

Note:* The mortality of snails exposed to WPN compared with that of snails exposed to four active concentrations of LDS.

There was a significant difference between the snail mortality rates at 0.4 g/m^2^ LDS and WPN after 3, or 7 days powdering (*P* = 0.021, *P* < 0.001, Table 2). The results of the spraying and powdering tests indicated no significant difference in mortality after exposure to 0.6 g/m^2^ LDS and the WPN for 1, 3, and 7 days (Table 2). Snail mortality after exposure to 0.8, and 1.0 g/m^2^ LDS for 1, 3, and 7 days was higher in comparison to sprayed or powered WPN.

### 3.2. The Snail Mortality Rate by Molluscicide Concentration and Exposure Time in Different Molluscicide Methods

A binary logistic regression model was used to analyze the correlation between snail mortality and molluscicide concentration and exposure time (Table 3). In the immersion group, showed a higher snail mortality risk at 2, 3 days than 1 days (OR = 3.329, *P* < 0.001; OR = 19.320, *P* < 0.001; Table 3). In the spraying and powdering groups, indicated a higher snail mortality rate risk at 3, 7 days than 1 day (Table 3); The immersion test showed a higher snail mortality risk in 0.4, and 0.6 g/m^3^ LDS than that of WPN. In spraying group and powdering test, there was no significant difference in snail mortality between 0.6 g/m^2^ LDS and WPN (*P* = 0.983; *P* = 0.630; Table 3), however, it showed higher snail mortality was observed with 0.8, and 1.0 g/m^2^ LDS in comparison to WPN.

**Table 3:** Analyzing the influence factors of the mortality of snails in different molluscicide methods by binary logistic regression.

Molluscicide Method	Variables	Estimate	Std. Error	OR (95% CI)	*P*-value
Immersion	Intercept	1.843	0.066	6.317 (6.242, 6.390)	0.000
	Day				
	1	-	-	1.000	-
	2	1.203	0.076	3.329 (2.867, 3.865)	0.000
	3	2.960	0.156	19.320 (14.219, 26.251)	0.000
	molluscicide				
	0.2 LDS	−0.229	0.084	0.795 (0.674, 0.938)	0.006
	0.4 LDS	0.209	0.092	1.233 (1.030, 1.476)	0.022
	0.6 LDS	0.757	0.106	2.132 (1.733, 2.622)	0.000
	1.0 WPN	-	-	1.000	-
Spraying	Intercept	1.268	0.043	3.555 (3.267, 3.866)	0.000
	Day				
	1	-	-	1.000	-
	3	0.569	0.040	1.767 (1.635, 1.909)	0.000
	7	1.164	0.046	3.204 (2.927, 3.507)	0.000
	molluscicide				
	0.4 LDS	−0.306	0.052	0.736 (0.665, 0.815)	0.000
	0.6 LDS	−0.001	0.054	0.999 (0.898, 1.111)	0.983
	0.8 LDS	0.221	0.057	1.247 (1.116, 1.393)	0.000
	1.0 LDS	0.277	0.057	1.319 (1.179, 1.475)	0.000
	1.0 WPN	-	-	1.000	-
Powdering	Intercept	0.934	0.039	2.544 (2.357, 2.747)	0.000
	Day				
	1	-	-	1.000	-
	3	0.556	0.036	1.744 (1.625, 1.873)	0.000
	7	1.171	0.041	3.225 (2.976, 3.496)	0.000
	molluscicide				
	0.4 LDS	−0.206	0.048	0.814 (0.742, 0.894)	0.000
	0.6 LDS	0.024	0.049	1.024 (0.930, 1.128)	0.630
	0.8 LDS	0.263	0.051	1.300 (1.178, 1.436)	0.000
	1.0 LDS	0.431	0.052	1.539 (1.389, 1.704)	0.000
	1.0 WPN	-	-	1.000	-

### 3.3. Cost Comparison

The costs of the recommended applications concentrations of LDS and WPN were compared. The recommended active concentration of LDS for immersion was 0.4 g/m^3^; it was 0.6 g/m^2^ for both spraying and powdering. In all cases, the cost of applying LDS was lower than that for applying WPN. Relative to WPN cost, application of LDS by immersion could reduce costs by 36.84% and application by spraying or powdering could reduce costs by 5.26% (Table 4).

**Table 4:** Cost comparisons between LDS and WPN at the recommended application concentration.

Content	50% WPN	10% LDS	Cost Reduction by Use of LDS Relative to Cost of WPN (%)
Commercial price of molluscicide (CNY Yuan/kg)	38.000	12.000	-
Recommended immersion concentration (Kg/m^3^)	0.002	0.004	-
Market price of application by immersion (CNY Yuan/ m^3^)	0.076	0.048	Decline 36.842
Recommended spraying or powdering concentration (kg/ m^2^)	0.002	0.006	-
Market price of application by spraying or powdering (CNY Yuan/ m^2^)	0.076	0.072	Decline 5.263

Note: Recommended concentration WPN = Recommended active concentration WPN÷50%. Recommended concentration LDS = Recommended active concentration LDS÷10%.

## 4. Discussion

In China, snail control is an important strategy for schistosomiasis control, and mollusciciding is the most widely used methods for snail control. Since the 1950s, more than 2000 chemicals have been screened for mollusciciding properties by Chinese scientists [30]. The chemical molluscicides, such as sodium pentachlorophenate, nicotinanilide, and bromoacetamide, have been in use since the 1950s [31]. However, these molluscicides are toxic to non-target animals and cause environmental pollution [32,33,34]. More than 50 plant species, of the 1000 or so that have been tested, have been confirmed to contain components with molluscicidal activity against *O. hupensis* [35]. Unfortunately, some plant molluscicides have low efficacy and/or high toxicity towards fish or other non-target animals [36]. In recent years some molluscicides have been reported as being effective in molluscicides have been reported as being effective and less toxic, such as the suspension concentrate of niclosamide [37], a potential molluscicide derived from the plant *Solanum xanthocarpum* [36], *Piplartine* against *Biomphalaria glabrata* [38], and some compound molluscicides [39,40].

LDS was recently developed in China to reduce the problems associated with WPN use. According to “Toxicological test methods of pesticides for registration” the national standard of the People’s Republic of China (GBl5670—1995), the acute LDS toxicity test was carried out via mouth, skin and inhalation with Wistar rats. Meanwhile, the eye and skin stimulating test with rabbit as well as skin stimulating tests with guinea pigs were also conducted. The results showed that LDS was weakly toxic to Wistar rats; and had no acute stimulation to rabbit skin and eye. The guinea pig skin sensitization test showed that it was a weak sensitizing substance [26]. In a comparison of WPN and LDS toxicity to fish, after 24 h of exposure to 0.4 g/m^3^ of WPN, fish mortality was 100%, whereas exposure to the same concentration of LDS resulted in 40% fish mortality. The 24-h median lethal concentration (LC_50_) of LDS to fish is 0.5657 g/m^3^ (mg/L). The LC_50_ of WPN to fish is 0.2049 g/ m^3^ (mg/L), which is 1.7 times that of WPN [23]. With the concentration of 0.4 g/ m^3^ (mg/L) for 72 h, the fish death rate using LDS and WPN was 50.0% and 100% respectively [24]. After exposure for 1, 6, 24, and 48 h to 0.25 g/ m^3^ (mg/L) of LDS or WPN, the up-climbing rates of snails in LDS were 62.2%, 50.0%, 42.2%, and 32.2%, respectively, whereas in WPN, the up-climbing rates were 70%, 56.7%, 48.9%, and 46.7%, respectively. This indicates that *O. hupensis* did not escape from LDS more easily than from WPN [24].

In immersion, the results indicate that there wasn’t a significant difference between the snail mortality rates of 0.4 g/m^3^ LDS and WPN after 1 and 3 days. In spraying and powdering, the molluscicdal effect of 0.6 g/m^2^ LDS is the same as that of WPN. 0.4 g/m^3^ LDS applied using the immersion method or 0.6 g/m^2^ LDS applied by spraying and powdering achieved the same molluscicidal effect as WPN. In our study, by using different methods, the snail mortality rates of molluscicide groups were related to exposure time and concentration respectively; it increased with concentration and exposure time. If the recommended application active concentration of LDS for immersion was 0.4 g/m^3^, and was 0.6 g/m^2^ for both spraying and powdering, the cost of applying LDS was lower than that for applying WPN, moreover, LDS is simpler to manufacture than WPN, and the production cost of LDS is about 10% less than that of WPN [23,25].

In this study, we tested the efficacy of LDS applied using three different methods. In China, different molluscicide application methods are typically used in different snail breeding environments. Immersion is used in irrigation ditches and drainage ditches, and ponds in inner embankments areas, in which the water levels are easy to control. Spraying is a commonly used method of molluscicide application, especially in environments where application by immersion is impractical [22]. Powdering is used in areas where there is a lack of water in the environment, such as mountainous areas [41]. LDS is formulated as a wettable powder, as is WPN. In some environments that experience water percolation and that are flushed with running all year round, the molluscicidal effect may be reduced. Such areas may require repeated application of molluscicide, thus increasing the molluscicide costs [41,42]. To ensure the effectiveness of LDS in different environments, we are currently researching a slow-release formulation of LDS.

Two LDS patents have been obtained from the State Intellectual Property Office of the People’s Republic of China (ZL200610005300.4, ZL201010274645.6). The patents have already been transferred to companies for production. At the time of publication, 6 tons of LDS will have been produced and applied in two counties in Hubei Province in China that is a schistosomiasis japonica endemic area.

Our study had some potential limitations. The pH and organic matter content of soil, vegetation and sunlight may influence the effective concentration of molluscicide [43]. We observed the meteorological conditions in the field, but we did not analyze the possible effects of these environmental factors at the field sites. Therefore, we were not able to evaluate the influence of these factors on molluscicidal effects.

## 5. Conclusions

The recommended application of active concentration of LDS is 0.4 g/m^3^ when applied by immersion and 0.6 g/m^2^ when applied by spraying or powdering. The cost of LDS is less than that of WPN. Therefore, LDS is a molluscicide suitable for application in schistosomiasis japonica epidemic areas.
